# *Classopollis* works as a significant indicator for the Cheirolepidiaceae paleovegetation arid zone, as proven by fossil records from Egypt

**DOI:** 10.1371/journal.pone.0318867

**Published:** 2025-03-25

**Authors:** Ahmed Maher

**Affiliations:** Department of Geology, Faculty of Science, Al-Azhar University, Assiut, Egypt; Birbal Sahni Institute of Palaeosciences: Birbal Sahni Institute of Palaeobotany, INDIA

## Abstract

This paper presents an investigation that explores two distinct areas relatable to paleogeographic reconstruction. These areas include the Matruh Basin in the Northwestern Desert and the October Field in the Gulf of Suez in Egypt. The application of Scanning Electron Microscopy (SEM) has exposed the unique morphological attributes of *Classopollis* Pflug 1953, so simplifying improved understanding of new paleoecological interpretations relevant to the Mesozoic era. The *Classopollis* Pflug 1953 assemblage derived from four examined wells reveals significant similarities and is indicative of a uniform vegetative cover characterized by the Cheirolepidiaceae ecological zone. The results infer that the *Classopollis* Pflug 1953 assemblage may be a basis for biostratigraphic correlation within the coastal arid regions that adjoin the Tethys Sea. The genus *Classopollis* Pflug 1953 appears as a representative of the arid belt of the early Mesozoic and explains the expansion of the Cheirolepidiaceae family in coastal and desert areas, which suggests various adaptive strategies employed by this family. This study expresses the phylogenetic interconnections among the Cheirolepidiaceae, Tomaxellia, and *Brachyphyllum* while synchronously explaining the ecological effects and morphological evolution associated with *Classopollis*.

## 1. Introduction

The risk of heated climatic conditions could be responsible for the death of organisms and cause global risk on the Earth [1]. The Cheirolepidiaceae conifers existed from the Triassic to the Late Cretaceous or possibly to the lower Tertiary period with significant geography [2]. The ecological information about the Cheirolepidiaceae can be known from its fossil remnants [3]. This plant family (Cheirolepidiaceae) played a key role in the Northern Hemisphere’s swamp ecosystems at lower to mid-paleolatitudes [4]. The identification of the dispersed *Classopollis* Pflug 1953 pollen grain to a certain species is always difficult [5].

The dispersal of *Classopollis* Pflug 1953 has been observed globally in Mesozoic rock layers [6]. The *Classopollis* Pflug 1953 pollen is widely distributed in the Jurassic and the Cretaceous deposits [7]. The xerophytic *Classopolli*s Pflug 1953, and *Ephedripites* Bolchovitina and Potonié 1958, pollen grains are considered climatic indicators [8]. The plants that produced *Classopollis* Pflug 1953, appearing in the Late Triassic period, spread across land areas surrounding the Tethys and the equatorial sea, and their remains have been found worldwide [9]. The parent plant of *Classopolli*s Pflug 1953 is versatile and capable of thriving in various environments. It can grow in coastal areas, dry regions with low rainfall and quick drainage, and arid, hot climates where salt rocks, gypsum formations, and red beds are present. Additionally, it can adapt to humid climates, flourishing in ridge hillocks and swampy areas [10].

In [11], the genus *Classopollis* describes a unique type of pollen grain from Mesozoic deposits in Europe. [12] detailed the germination process, noting a distal operculum about 20 μm in diameter. [13] reported that the generic name *Tetradopollenites* Sittler 1954 is a homonym of *Tetradopollen*ites Pflug and Thomson 1953, and this individual Sittler use by [14] as a new infraturma for *Classopollis* type grains is inappropriate. [15] emphasized the taxonomic importance of these grains due to their complex features. [6] confirmed the genus’s validity, highlighting features like a circumpolar canal, thickened equatorial band, distal cryptopore, and proximal tetrad scar. [16] identified different morphotypes, including *C*. *classoides* and *C*. *torosus*, and described a new genus and species, *Circumpolles*, characterized by an operculum-like structure and numerous Ubisch bodies. [17] conducted an ultrastructural analysis of the exine to explore the evolutionary origins of the Cheirolepidiaceae, which produce *Classopollis* pollen.

The present research aims to recognize and study the ecological effects and distribution of *Classopo*llis Pflug 1953 pollen, which can help to explore its evolutionary line and biogeography and investigate potential factors contributing to its adaptation. The study focuses on two fields from different areas in Egypt containing Mesozoic strata, aiming to understand the environmental conditions and expansion of the Cheirolepidiaceae plants based on their fossil pollen.

## 2. Geologic settings

**Fig 1:**
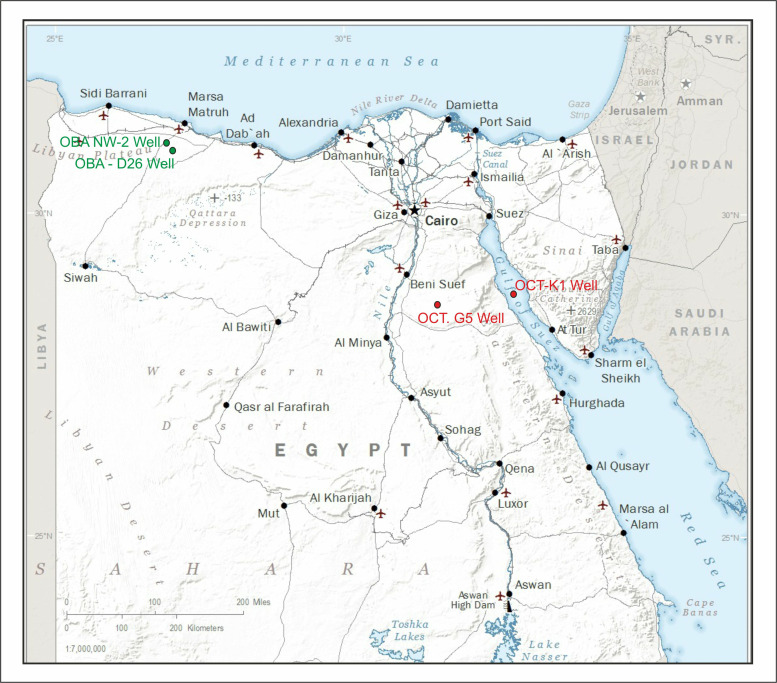
The location map of the studied locations includes the OBA-D26 and OBA NW-2 wells, the northern Western Desert in green dots, and the OCT. G.5 OCT-K1 in the Gulf of Suez wells in red dots in the Northwestern Desert of Egypt. Map drawn and modified from using Generic Mapping Tools [102].

The study focuses on two different geographic areas in Egypt: the first is in the Gulf of Suez in the October Field and the second in the Obayied Field in the Northwestern Desert (Fig 1). The four study wells (Fig 1) are represented by two red dots used in the Gulf of Suez and the green dots used in the Northwestern Desert, and the star indicates the location of Cairo City in Egypt.

### 2.1. The first location of the present study ( Fig 1 ) in the Gulf of Suez

The Gulf of Suez is considered to represent the borderline between the African plate and the Sinai microplate [18], and it is segmented into three primary depocenters [19]. Three main depositional periods are recognized in these basins: pre-rift, syn-rift, and post-rift [20]. The Gulf of Suez has experienced a complex tectonic history [21]. These tectonic movements have affected the areas of the Tethyan regions [22]. In the Gulf of Suez, there is a thick sequence of undifferentiated Paleozoic-Mesozoic Nubia sandstone [23]. The Neotethys Sea was established during the Permian to the Jurassic ages to be extended from Oman to Iran, Iraq, the Mediterranean Sea, and Egypt [20].

The Nubia sandstone involves extensive marine sandstone from various environments and continental settings [23]. The Nubia Sandstones are situated in the southern region of the Gulf of Suez, are complex and not differentiated [24–25], encompassing Nubia A (lower Cretaceous?), Nubia B (Carboniferous), and Nubia C and D (lower Paleozoic) [18]. The samples were collected from the October Field and targeted the Nubia sandstone sediments (Figs 4 and 5).

**Fig 2:**
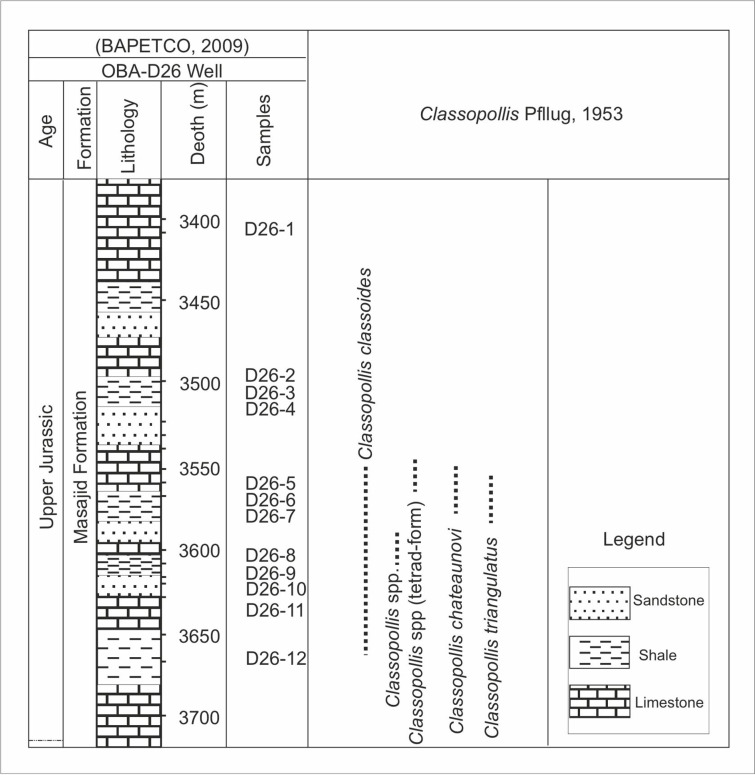
Lithologic log of the studied interval of the Masajid Formation and occurrences of the recorded species of the *Classopollis* Pflug 1953 pollen in the OBA-D26 Well, northern Western Desert, Egypt, based on [31].

**Fig 3:**
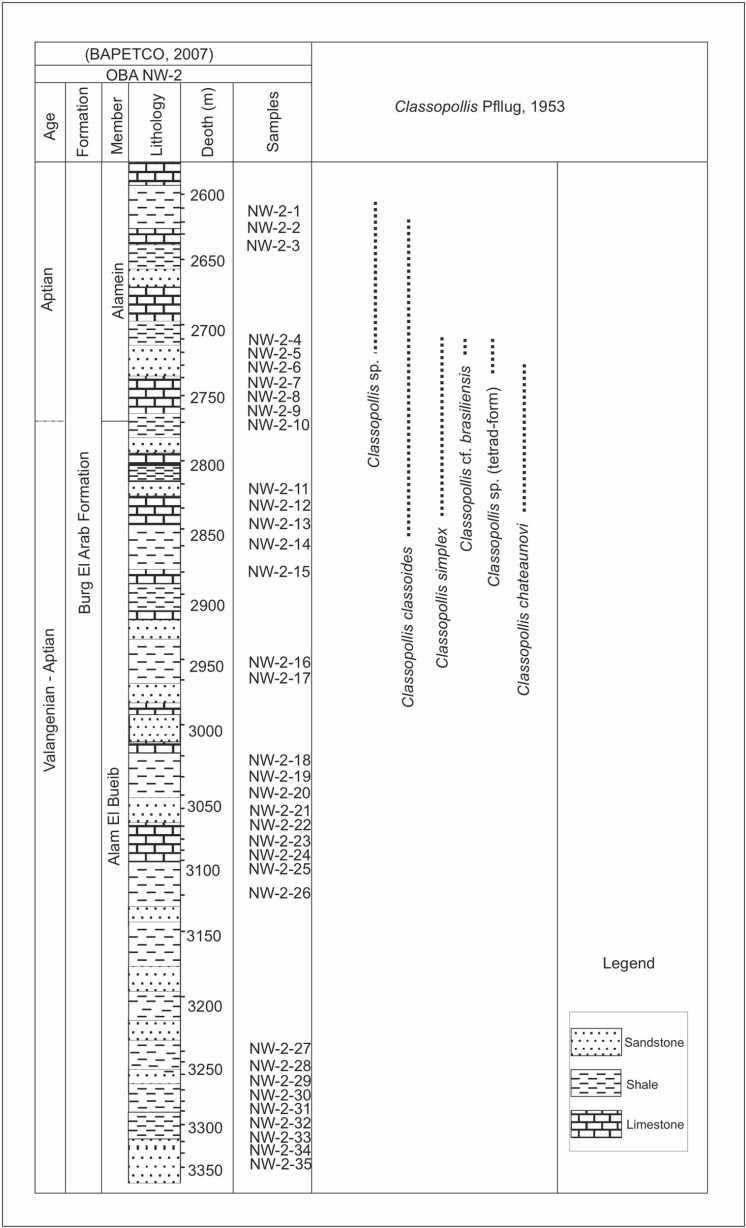
Lithologic log of the studied Alam El Bueib and Alamein members and occurrences of the recorded species of the *Classopollis* Pflug 1953 pollen in the OBA NW-2 Well, northern Western Desert, Egypt (based on 32).

### 2.2. The second location of the present study (Fig 1) in the Northwestern Desert

The region of the northwestern desert represents an essential part of the African plate, and it represents marine Mesozoic transgression in northern Egypt; it is aligned with the beginning of the Neotethys [23; 26] and caused the formation of abundant sedimentary basins and is marked by the accumulation of fluvial continental [27]. This transgressive cycle [26] led to the accumulation of Alamein carbonates in a restricted marine and lagoonal setting [27]. The Obaiyed Field is in the northern basins near the Marsa Matruh (Fig 1).

The lower Cretaceous period in the Northwestern Desert is exhibiting a transgressive cycle involving the deposition of fluvial continental sediments (Neocomian), succeeded by transitional, near-shore deltaic sediments from the Aptian and Albian epochs [27]. The Burg El Arab Formation from the lower Cretaceous is composed of a sequence of clastic rocks spanning from fine to coarse-grained [26]. This formation can be segmented into the Alam El Bueib and Kharita members, separated by a carbonate unit composed of the Alamein and Dahab members [26]. The samples were collected from the Obayied Field and targeted the Alam El Bueib and the Alamein members (Figs 2 and 3).

## 3. Materials and methods

A total of 49 and 37 ditch-cutting samples from the October and Obayied fields were prepared for palynology, respectively. The kerosene was used and succeeded by a soap solution to eliminate contamination of the drilling process. The samples (5–7 g) were treated with cold hydrochloric acid (HCl-HF), followed by sieving through 10 μm meshes, without oxidation, by standardized palynological techniques [28]. Coverslips were applied on slides using ICL’s Elvacite® 4345 acrylic resin dissolved in limosol.

An Olympus BX50 equipped with a computerized digital camera was employed for transmitting light microscopy. Some samples were selected for analysis and showed adequate fluorescence for subsequent investigations; they responded when stimulated by WBV, WIB, and WU radiation. SEM observations were mounted on aluminum stubs and coated with platinum using a magnetron sputter coater (JUC-5000, JEOL, Tokyo, Japan) and observed under SEM (JSMIT100, JEOL). Some selected samples are employed to fabricate a petrographic thin section and are observed under light microscopy. All residues documented in this research are currently stored in the Department of Geology at Al-Azhar University (Assiut). All data are within the manuscript, and materials are available in the Department of Geology, Faculty of Science, Al-Azhar University, 71524 Assiut, Egypt.

### 3.1. The four wells used in the current study

The October Field is the first location used in the present study (Fig 1), located in the central Gulf of Suez, which is defined as a half-graben and is considered one of the deepest and largest basins in the area [23]. The October field is the third largest oil field in Egypt, producing over 420 million bbl of oil from its discovery in 1977 until January 1991. It is the northernmost giant oil field in the Gulf of Suez rift basin [20]. In the current study, two wells are used from the October Field. The OCT. G.5 Well, which was drilled in 1989 [latitude 28, 49’, 18. 83” N & longitude 31, 41, 11. 29” E] [29], a total of 26 cutting samples were obtained from the interval of (3435. 096–3813. 048 m) from the OCT. G.5 Well. The OCT-KI Well was drilled in 1996 for developmental purposes [latitude 28, 49’, 45. 20” N & longitude 33, 04, 59. 86” E] [30], samples were specifically retrieved from the Nubia sandstone from the shale intervals. A collection of 23 ditch-cutting samples was acquired from the interval of (depth 3383. 28–3718. 56 m) from the OCT-K-1 Well.

The Obayied Field is the second location used in the present study, which lies in the Western Desert of Egypt near the Marsa Matruh (Fig 1), around 450 km away from Cairo and 50 km to the south of the Mediterranean coast [27]. This Obayied field is located roughly 450 km west of Cairo and approximately 50 km south of the Mediterranean coast (Fig 1). Specifically, the investigation focused on the OBA-D26 Well (latitude 31°, 06’ 33. 66” & longitude 26°, 38’, 55, 725” E) [31] and the OBA NW-2 Well (latitude 31°, 10’ 65. 819” & longitude 26°, 32’, 43, 948” E) [32].

The samples selected for the palynology analyses were obtained from the shale intervals in both the October and Obayied fields, respectively. The samples were investigated for lithology under a binocular microscope. The samples collected from the October Field were ditch-cutting and were composed of clastic sediments containing shale, carbonate, and fragments of plant remains; there are rare ichnofossils observed. However, the samples obtained from Obayied Field are composed of very dark black shale, and there are findings of foraminifera that might be Orbitolinids observed.

## 4. Results

**Fig 4:**
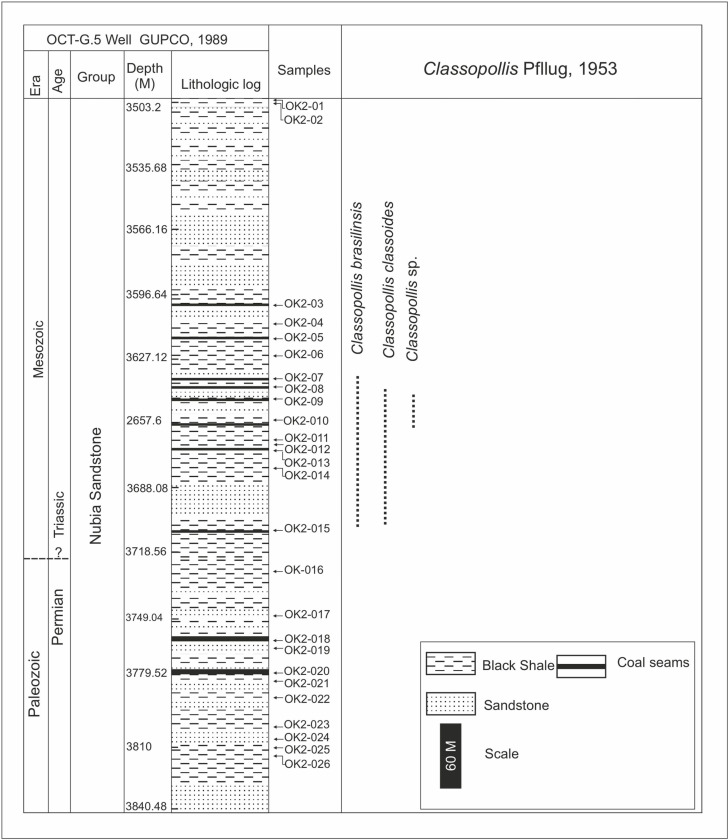
Lithologic log and occurrences of the recorded species of the *Classopollis* Pflug 1953 pollen in the OCT. G.5 well, Gulf of Suez, Egypt, based on [29].

**Fig 5:**
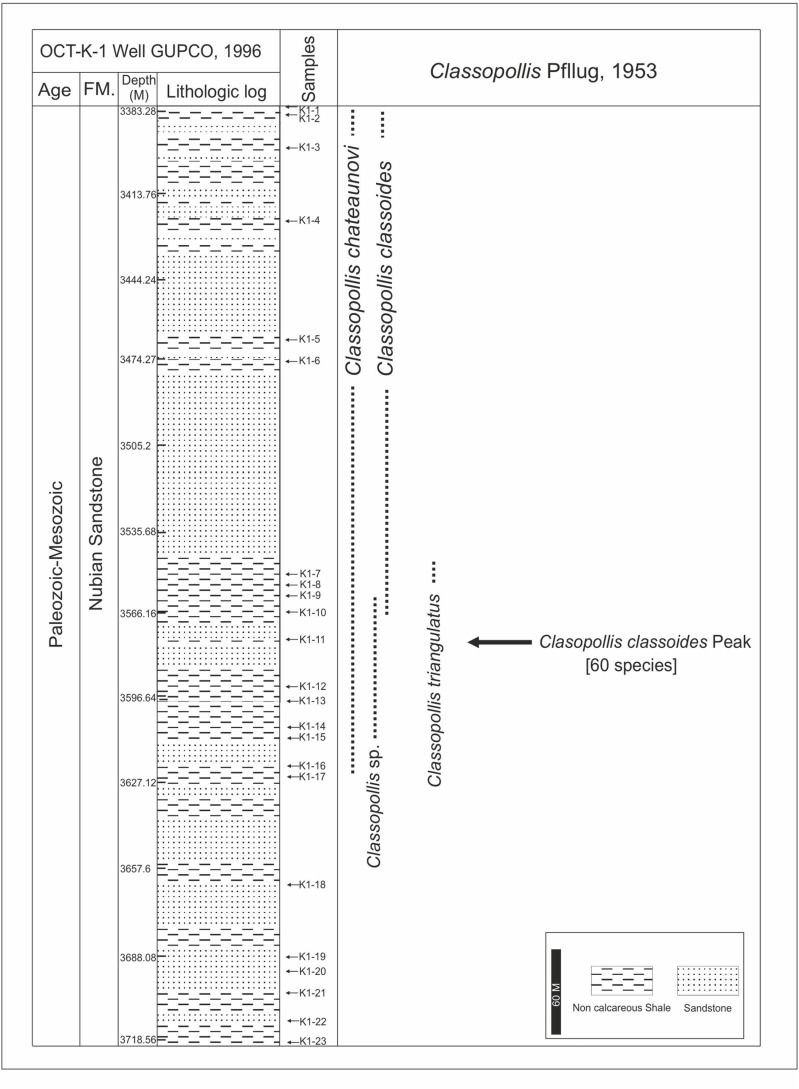
Lithologic log and occurrences of the recorded species of the *Classopollis* Pflug 1953 pollen in the Nubia Sandstone in the OCT-K-1 well, Gulf of Suez, Egypt (log based on 30).

The eighty-six samples collected from the four wells are used to isolate *Classopollis* Pflug 1953 from the palynological data. In the studied samples from the Western Desert, the *Classopollis* Pflug 1953 is obtained from the Alam El Bueib Member of the Burg El Arab Formation from the Barremian-Aptian age and the Masajid Formation of the Upper Jurassic age (Figs 2 and 3). In the studied samples from the Gulf of Suez, *Classopollis* Pflug 1953 is obtained from the upper interval of the Nubia Sandstone from the Nubia “B” Member (Figs 4 and 5). The *Classopollis* Pflug 1953 assemblage shows well-preserved conditions (Figs 7–11). The first appearance and last appearance are illustrated in (Figs 2–5).

**Fig 6:**
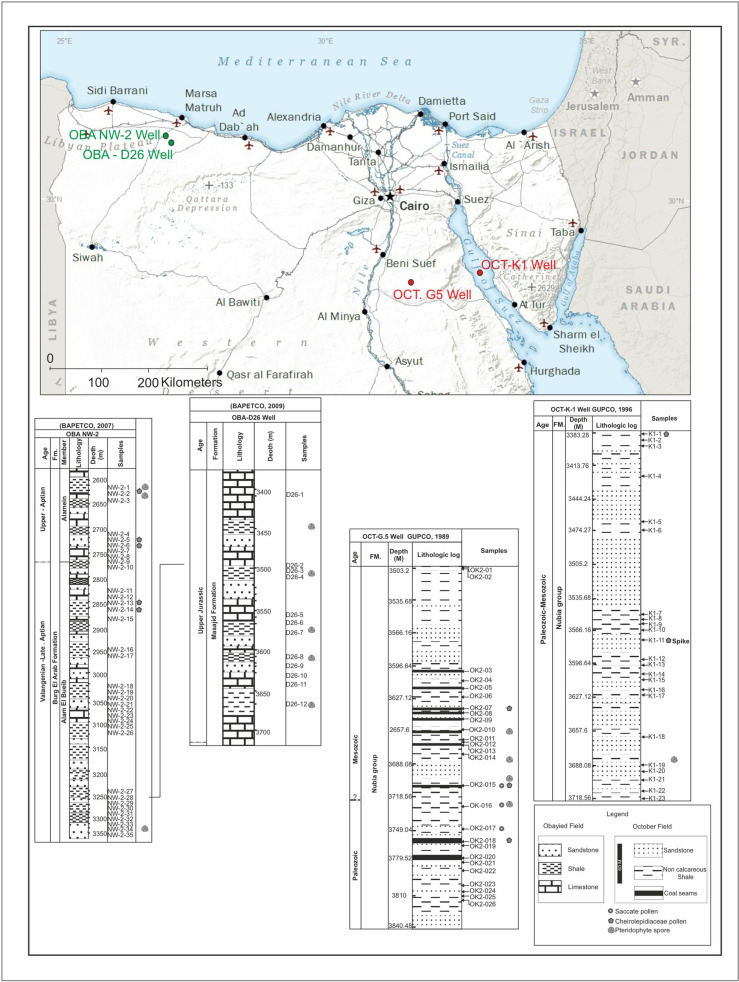
The connection between the four studied wells represented October and Obayied fields, respectively, with their location in the map (Map drawn and modified using Generic Mapping Tools from 102) following the journal instructions for the maps. The stratigraphic distribution of the *Classopollis* Pflug 1953 and their parent plants (Cheirolepidiaceous) are added.

**Fig 7:**
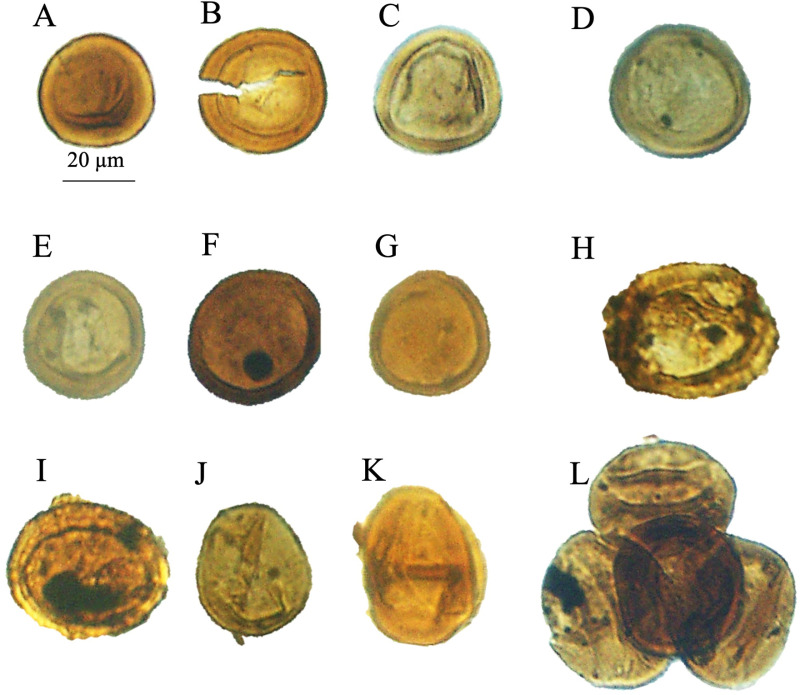
Recorded *Classopollis* Pflug 1953 in the Obayied Field. A-G. *Classopollis* simplex (Danzé-Corsin & laveine) Reisser and Williams 1969; A. NW-2-1. B. NW-2-5. C. NW-2-13. E. NW-2-12. F. NW-2-1. G. NW-2-11. H. *Classopollis classoides* Fensome 1983. I-K. *Classopollis* spp. L. *Classopollis-*tetrad. L. NW-2-5-A1. All samples from the OBA NW-2 Well.

**Fig 8:**
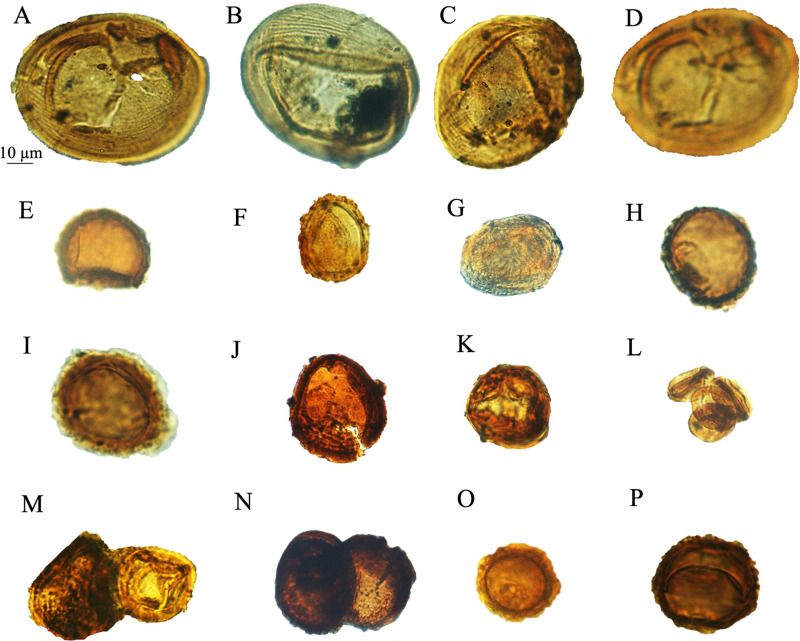
Classopollis Pflug 1953 in the October Field. A-D. *Classopollis brasiliensis* Herngreen 1975. E, O, P. *Classopollis* sp. H, I. *Classopollis classoides* Fensome 1983. G, J, L, N. *Classopollis* spp. F, K, M. *Classopollis chateaunovi* Reyre 1970 were extracted from the OCT-G.5 and OCT-K-1 wells, respectively.

**Fig 9:**
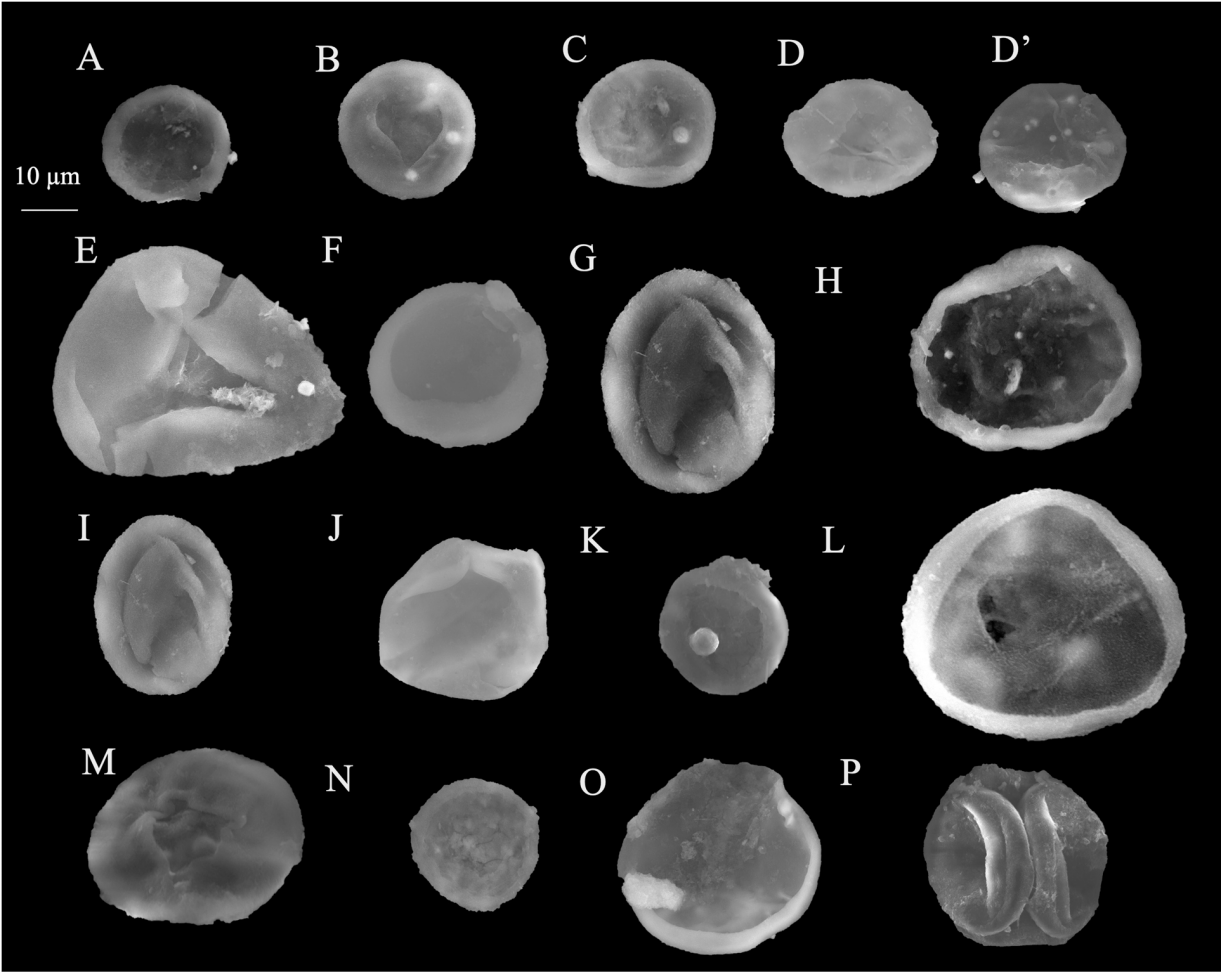
SEM microspores of Obayied Field. A, C, F, K, N. *Classopollis* cf. *simplex*. B. *Classopollis simplex* (Danzé-Corsin and Laveine 1963) Reiser and Williams 1969*.* E. *Deltoidospora* sp.; NW-01. G, I, J. *Classopollis chateaunovi* Reyre 1970. H. *Balmeiopsis limbatus* (Balme) Archangelsky 1979; OBA-D26 Well. D, D’. L, M. *Classopollis classoides* Pflug 1953, emend. Pocock and Jansonius 1961. O. *Classopollis* sp. P. *Classopollis* spp. were extracted from the OBA-D26 and OBA-NW-2 wells, respectively. A-P; OBA-D26 Well.

**Fig 10:**
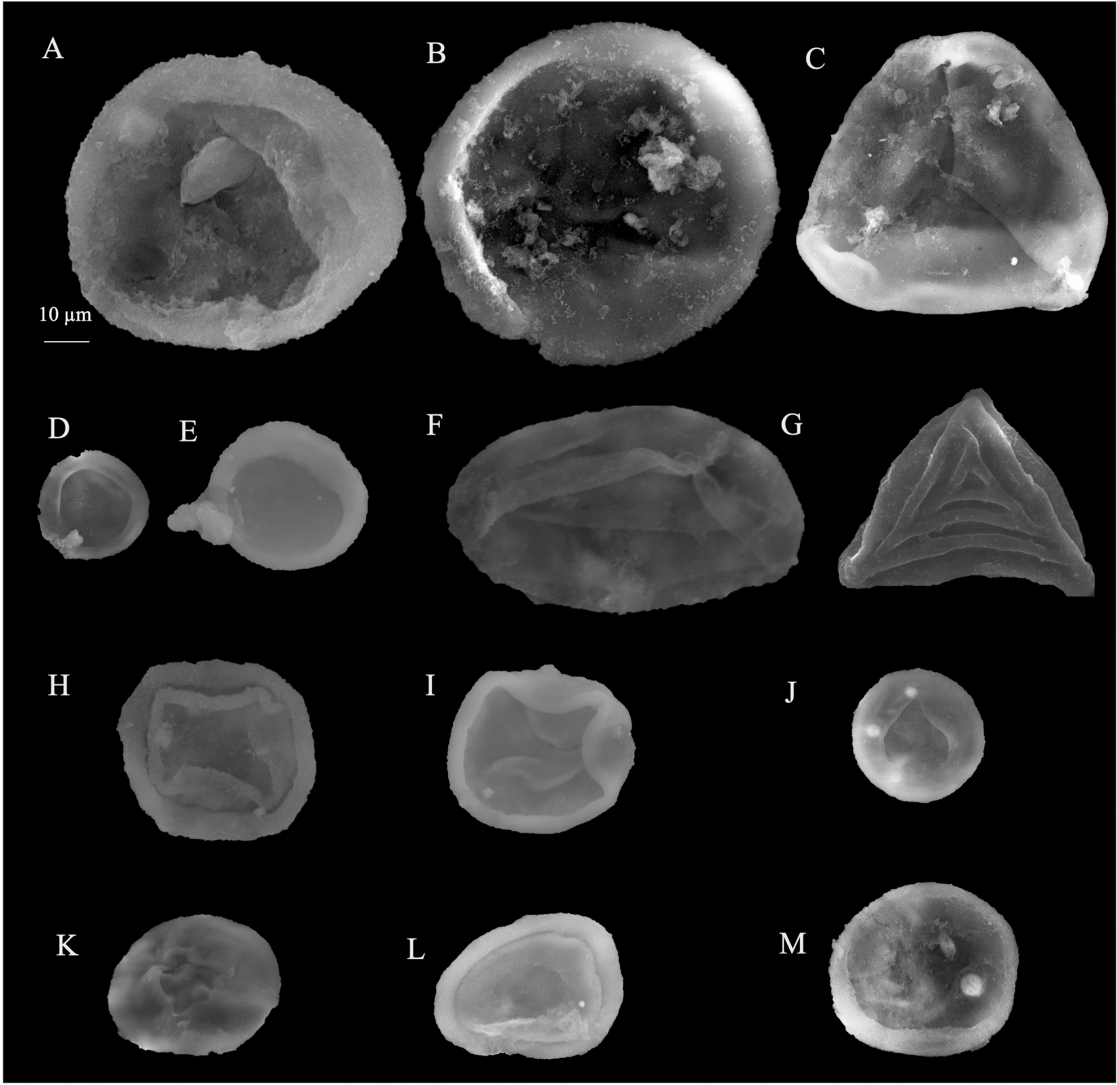
SEM microspores of Obayied Field. A, B. *Classopollis* spp. C. *Deltoidospora psilostoma* Rouse 1959; OBA-D26 Well. F. *Classopollis chateaunovi* Reyre 1970. D, E. *Classopollis* cf. *simplex* (Danzé-Corsin and Laveine 1963) Reiser and Williams 1969. A. OBA-D26 Well. F. OBA-D26 Well. G. *Cicatricosisporites* sp.; OBA-D26 Well. H-I. *Classopollis chateaunovi* Reyre 1970. J-K. *Classopollis classoides* Pflug 1953, emend. Pocock and Jansonius 1961*.* L. *Classopollis meyeriana* (Klaus 1960) de Jersey 1973. M. *Classopollis* sp. were extracted from the OBA-D26 and OBA-NW-2 wells, respectively.

**Fig 11:**
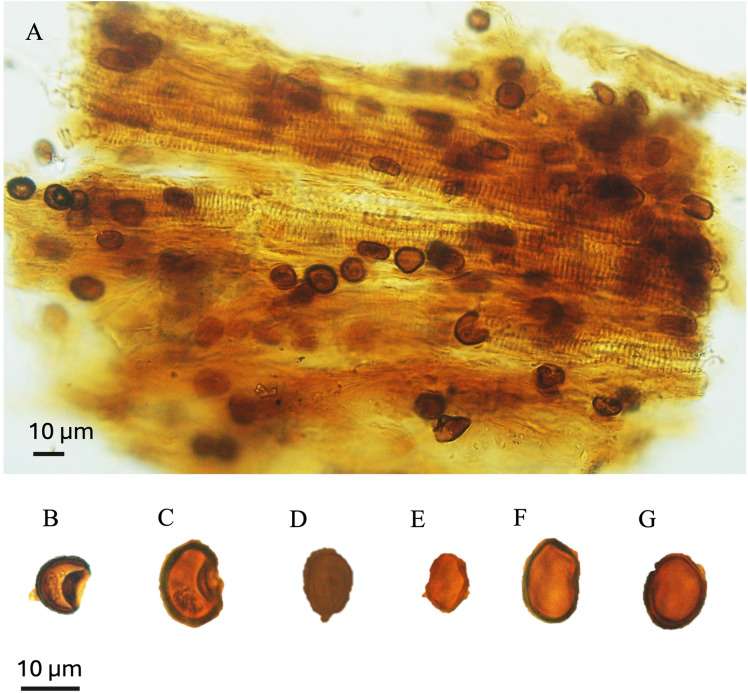
Isolated cuticle from the October Field. **A.** Cuticle dominated with *Classopollis classoides*/*simplex* complex. B-G. Isolated species from the surface of the cuticle were extracted from the OCT-G.5 and OCT-K-1 wells, respectively.

The study recognized two *Classopollis* Pflug 1953 assemblages from the two studied fields. A table list of the species describing the differences between species of the *Classopollis* Pflug 1953 is established.

There are some selected samples from both studied October and Obayied fields that are investigated for the petrographic studies. The results of the October Field samples are composed of gypsum, iron oxides, and evaporites (Fig 13; A), while another is observed to be dominated by mineral inclusions (Fig 13; B). The results of the Obayied Field samples are composed of ooids of unknown origin with iron oxides and containing Orbitolinids (Foraminiferida) (Fig 13; C-D).

**Fig 12:**
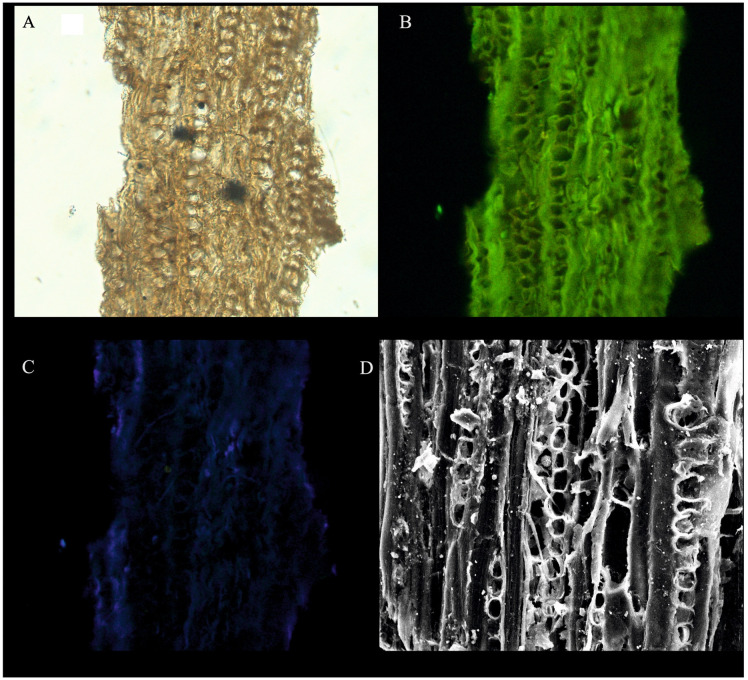
Isolated cuticle from the October Field. A-D, Cucicle. A-D. The cuticle shown layered of different cells of cf. *Frenelopsis* Schenk 1869 cuticle plants were extracted from the OCT-G.5 and OCT-K-1 wells, respectively.

### 4.1. *Classopollis* Pflug 1953 in the October Field

The *Classopollis* Pflug 1953 was recorded from the OCT. G.5 Well (Fig 4) in the interval from [3627.12–3749. 04 m] and is composed of *Classopollis brasiliensis* Herngreen 1975 [33] (Fig 8 A-D), *Classopollis classoides* Fensome 1983 [34] (Fig 8; H, I), *Classopollis* spp., (Fig 8; G, J, L, N) and *Classopollis chateaunovi* Reyre 1970 [5] (Fig 8; F, K, M). A peak of the *Classopollis classoides*/*simplex* complex is observed in numerous preserved sheets of cuticles (Fig 11), some of them isolated (Fig 11; B-G). The microflora of the Nubia sandstones is composed of *Classopollis* spp. (Fig 11), and was noted with several dominance of well-preserved SEM cuticle and fluorescence (Fig 12).

The distinguishing feature of the microflora content of the October Field is that it is composed of diverse Triassic monosaccate and spore assemblages, but the current work focuses only on *Classopol*lis Pflug 1953 assemblages. This *Classopollis* Pflug 1953 is associated with palynoflora of the latest Triassic age (Fig 14; A-F). Some of the associated microflora observed in the studied October Field samples are *Aratrisporites* sp., *Leiotriletes* sp., *Ovalipollis ovalis* (Krutzsch) Scheuring 1970, *Praecolpatites sinuosus* Balme and Hennelly 1956, *Tricolpites* sp., and *Kraeuselisporites cuspidus* Balme 1963 (Fig 14; F).

**Fig 13.**
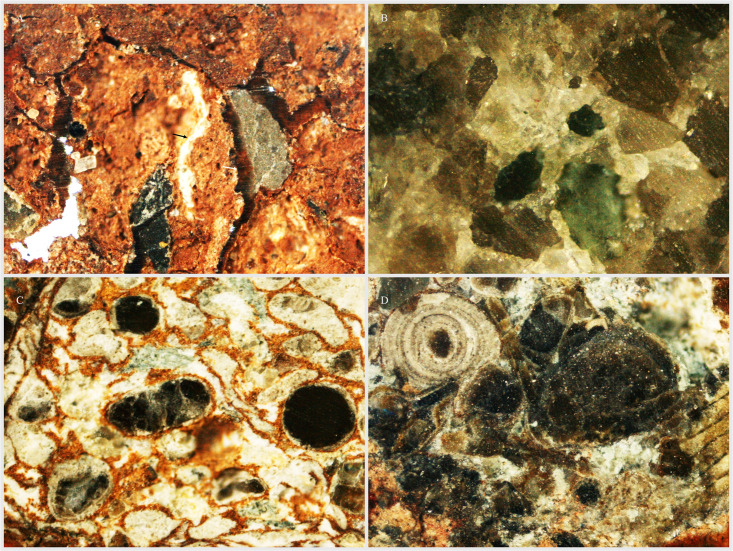
A.-B. Some represented petrographic samples from the October Field wells. A. Petrographic samples show gypsum (black arrow), and evaporites (white arrow). B. Represented petrographic samples containing unknown minerals. C-D. Some represented petrographic samples from the Obayied Field wells. C. Represented petrographic samples showing iron oxide materials as a matrix between the unknown ooids. D. Represented petrographic sample showing foraminifera.

**Fig 14:**
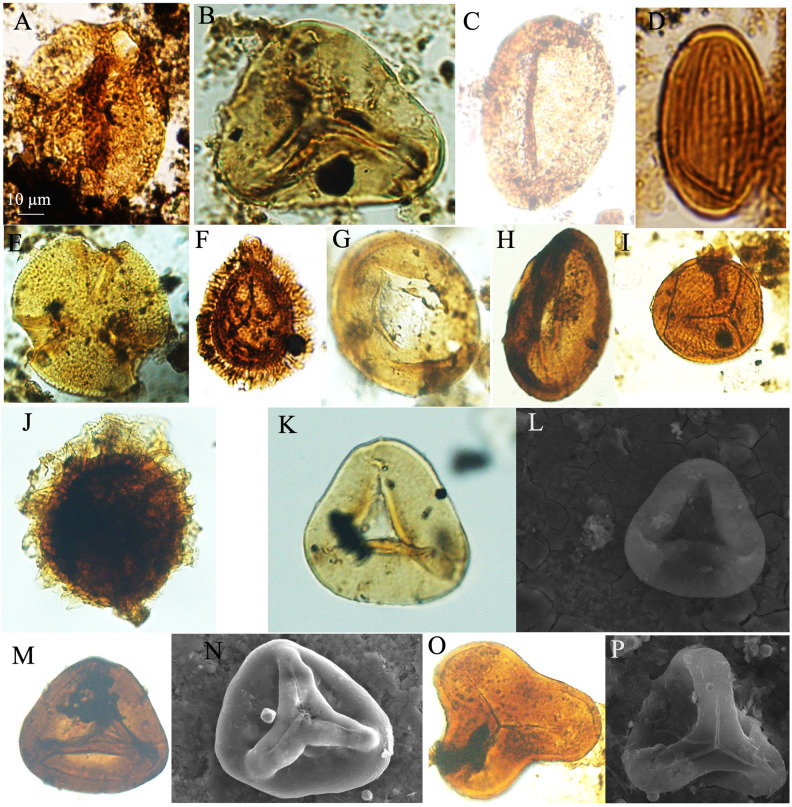
Selected represented microspores from the studied samples associated with the isolated pollen of the *Classopollis* Pflug 1953 under the present study. A-F. Microspores from the studied October Field samples. A. *Aratrisporites* sp. B. *Leiotriletes* sp. C. *Ovalipollis ovalis* (Krutzsch) Scheuring 1970. D. *Praecolpatites sinuosus* (Balme & Hennelly 1956). E. *Tricolpites* sp. F. *Kraeuselisporites cuspidus* Balme 1963. G-P. Microspores from the studied Obayied Field samples. G. *Balmeiopsis limbatus*. H. *Monocolpopollenites* sp. I. *Cicatricosisporites* sp. J. *Crybelosporites* sp. K-L. *Deltoidospora psilostoma* Rouse 1959. M-N. *Dictophyllidites harrisii* Couper 1958. O-P. *Concavissimporites punctatus* Pocock 1964.

### 4.2. *Classopollis* Pflug 1953 in the Obayied Field

The *Classopollis* Pflug 1953 was recorded in the Obayied Field from the OBA-D26 Well (Fig 2) in the interval [3550–3650 m] and the OBA NW-2 Well from the interval [2600–2850 m]. The isolated *Classopollis* Pflug 1953, from the microflora of the Obayied Field, are *Classopollis simplex* (Danze-Corsin and Laveine 1963) Reiser and Williams 1969 [35] (Fig 7; A-G), *Classopollis* spp., (Fig 7; I-K), *Classopollis chateaunovi* Reyre 1970 [5] (Fig 9; G, I, J), *Classopollis classoides* [11] (Fig 9; D, D’. L, M.), and *Classopollis*-tetrad (Fig 9; P).

The microflora content of the October Field is composed of diverse spore assemblages, but the current work focuses only on *Classopollis* Pflug 1953 assemblages. This microflora content in the October Field is mainly of the Barremian-Aptian age; some of the other represented microflorae are *Balmeiopsis limbatus* (Balme) Archangelsky 1979, *Monocolpopollenites* sp., *Cicatricosisporites* sp., *Crybelosporites* sp., and *Deltoidospora psilostoma* Rouse 1959; *Dictophyllidites harrisii* Couper 1958; and *Concavissimporites punctatus* Pocock 1964 (Fig 14; O-P).

### 4.3. Potential connectivity of the gymnosperm Cheirolepidiaceae between the two fields

The *Classopollis* Pflug 1953, observed in the OCT-G.5 and OCT-K-1 wells from the October Field and also from the OBA-D26 and OBA NW-2 wells from the Obayied Field, are similar based on the species level. *Classopollis classoides* Pflug 1953, and *Classopollis simplex* (Danze-Corsin and Laveine 1963) Reiser and Williams 1969 [35], which were observed in the October Field (Fig 11), are similar to the same observed in the Obayied Field (Fig 7). The detailed structure of the *Classopollis* Pflug 1953 was observed in the SEM (Fig 9 and 10). A correlation between the four studied wells is summarised in (Fig 6) by adding the parent producing the *Classopollis* Pflug 1953, including the affinity to the Cheirolepidiaceae plant.

### 4.4. Systematic palynology

**Genus** *Classopollis*

**Synonym**

1953 *Classopollis* Pflug, p. 91.
1953 *Circumpollis* Pflug, p. 92.
1953 *Canalopollis* Pflug, p. 93.
1954 *Tetradopollenites* Sittler, p. 339 (nom. Mud).
Non-1953 *Tetradopollenites* Pflug and Thomson in Thomson and Pflug, p. 112 [36].
1956 (1955) *Trachyaletes* Sah, p. 64 (nom. Mud.).
1961 *Classopollis* Pflug emend. Pocock and Jansonius, p. 443 [13].
1963 *Classopollenites* Danzé-Corsin and Laveine, p. 105.
1963 *Monilapollis* Chang, p. 439.
1963 *Pagiophyllumpollenites* Chang, p. 439.
1964 *Granuloperculatipollis* Venkatachala and Góczán, p. 219 [37].
1965 *Classoidites* van Amerom, p. 119.
1966 *Gliscopollis* Venkatachala, p. 99 [38].
1970 *Classopollis* Pflug emend. Reyre, p. 311. [5]
1976 *Classopollis* Srivastava [6]

***Classopollis chateaunovi*** Reyre 1970 (Fig 8; F, K, M.; Fig 9: G, I, J) [5]

**Description:** Grains are anisopolar, amb circular to oval, exine 1–1.5 μm, thicker in the equatorial region, and thinning towards the poles. Pore cavities occupy the grain. A similar tetrad mark and a subsequent canal are present. The proximal pole occasionally has a triangular area in which the exine is absent.

***Classopollis classoides*** Pflug 1953, **emend.** Pocock and Jansonius 1961 **[13]** (Fig 9: D, D’. L, M.)

**Description:** Grains are anisopolar, amb circular, and composed in a polar direction. Each pollen grain possesses a circular cryptopore on the distal polar area. No tetrad mark was observed. A central cavity that occupies the grain might have a biological role. Exine 1–2 μm thick in the equatorial region and thinning towards the pole.

***Classopollis simplex* (Danzé-Corsin and Laveine, 1963) Reiser and Williams 1969 [35]** (Fig 7: A-G; Fig 9: B)

**Description:** Grains anisopolar, amb circular. Exine 1–2 μm thick in the equatorial region. The proximal pole usually has a circular area. A distal pole is rarely characterized by the presence of a circular pore. A central cavity without a tetrad mark was observed.

***Classopollis brasiliensis* Herngreen 1975 [33]** (Fig 8: A-D)

**Description:** Grains anisopolar, amb rounded to oval, and striated ornamentation connects the poles. The proximal pole usually has an oval area. A central cavity without a tetrad mark surrounded by striations was observed.

***Classopollis* sp.** (Fig 7: I-K and 8: G, J, N).

*Classopollis torrosus* **Reissinger 1950** [**39**]; **Couper 1958**

**Description:** Grains anisopolar, with an amb circular, pore aperture in the proximal pole, the ring-shaped transition zone between the distal hemisphere and the equatorial band. Some grains are monoporate, and circular to subcircular, with exine 2–4 μm thick. Sculpture consists of minute granules and small folds in some specimens. Pollen, tetrahedral, and others are individual pollen.

***Classopollis meyeriana* (Klaus 1960)** [**14**] **de Jersey 1973** (Fig 10; L)

**Description:** Pollen grain has a two-layered exine. The inner exine is thicker than the outer one. Sculptured granulate. Cryptopore, tetrad mark, and canal observe. A central cavity was observed without a tetrad mark.

## 5. Results and discussions

The investigated *Classopollis* Pflug 1953, is attained from ditch-cutting samples, so downhole contamination is probable. The *Classopollis* Pflug 1953 [11] is used to reconstruct the botanical affinity and the correlation between the four studied wells. A correlation between the *Classopollis* Pflug 1953, assemblages and the ecological affinity of their parent plants has been attempted to inform on the paleoclimtological and paleoenvironmental reconstructions.

The *Classopollis* Pflug 1953 assemblage recorded from two studied locations of the October and Obayied fields represented distinctive pollen assemblages with a circular germinal furrow (Fig 10; H-L). The *Classopollis* Pflug 1953 is attributed to Cheirolepidiaceae [3]. *Classopollis brasiliensis* Herngreen 1975 [33] (Fig 8 A-D) closely resembles the *in situ* species found in the fossilized trees of *Protocupressinoxylon purbeckensis* Francis 1983 [40] in the Lower Purbeck (Upper Jurassic) layers of Dorset, England [40]. The cuticles (Fig 12) also show significant similarities to those of the same species depicted in Plate 39 [40]. Additionally, the *Classopollis* Pflug 1953, and associated dispersed cuticles from the October Field bear resemblances to species identified from the conifer *Pseudofrenelopsis parceramosa* (Fontaine) Watson 1977 [41]; from the Barremian of the Isle of Wight [42]. Further, the *Classopollis simplex* (Danzé-Corsin and Laveine 1963) Reisser and Williams 1969 [35] (Fig 7) share close similarities with the isolated species from the *Frenelopsis coniferales* of Cheirolepidiaceae from the Lower Cretaceous Berriasian-Barremian in Spain [43].

The *Classopollis* Pflug 1953, found in the Obayied Field (Fig 9) during the Barremian-Aptian, has significant similarities with the *Classopollis* Pflug 1953, isolated from the Cheirolepidiaceous conifer pollen cones (*Classostrobus arkansensis*) [44–45] from the Lower Cretaceous Aptian/Albian of the Holly Creek Formation in Arkansas [46–47]. Some of these conifer pollen cones were attached to *Pseudofrenelopsis parceramosa* Fontaine [46]. Additionally, there are notable resemblances with the *in situ Classopollis*-type pollen linked with *Frenelopsis ramosissima* Fontaine, with both exhibiting identical similarities (Fig 2; G) [48] and the current SEM species in (Fig 10; L).

The preservation of Cheirolepidiaceae cuticle is suggested to occur in fluvial flood basin environments resulting from small trees [3]. The Gondwana records of Cheirolepidiaceae by [3] include *Tomaxellia* Archangelsky 1963 [49–50], *Frenelopsis* Kunzmann [50–51], *Pagiophyllum* McLoughlin [52–53], and *Brachyphyllum* Gould [54–55]. The Cheirolepidiaceae may include plants with diverse habits, ranging from small shrubs to trees, and they dominate various environments [43–47]. The diversity in the recorded *Classopollis* Pflug 1953, from the Egyptian sediments in the current study, could have witnessed the evolution of the Cheirolepidiaceae in the Tethys areas. The variations in pollen grain structure (Table 1) and shown in (Figs 9 and 10), such as exine thickness and pore structure, can help identify plants’ evolutionary adaptations for the first time.

**Table 1:** Table list of the species covered in the text, describing the differences between species, to demonstrate well the criteria on which the authors base their identification.

Species	Author systematic remarks	Description (current study): Main differences between the species of *Classopollis* Pflug 1953 [11]
*Classopollis chateaunovi* Reyre 1970 [5]	**Grain Structure**: Anisopolar, circular to oval, initially spherical or ovoid but often compressed along the polar axis. **Exine**: 1–1.5 µm thick, thicker in the equatorial region, tapering toward the poles. Uniformly distributed, diminutive verrucae ( < 0.5 µm), often fused, forming an incomplete pseudoreticulum. **Equatorial margin** ~ 8 µm wide. No rimula detected. **Poles**: the proximal pole may have a triangular area (sides up to 6 µm) where exine is absent. Distal pole has a circular pore (5–12.5 µm in diameter) [56; 3].	*Classopollis chateaunovi* Reyre 1970 [Fig 8; F, K, M.; Fig 9: G, I, J) [5]. **Structure**: Anisopolar, amb circular to oval. **Exine**: 1–1.5 µm thick, thicker in the equatorial region, thinning towards the poles. **Key Features**: Pore cavities occupy the grain, similar tetrad mark, subsequent canal present. **Proximal Pole**: Occasionally has a triangular area where the exine is absent.
*Classopollis classoides* Pflug 1953, emend. Pocock and Jansonius 1961 [13]	**Pollen Structure**: Tetrahedral tetrads, spherical or corn-shaped. **Key Features**: Circular cryptopore on distal polar area, trifid tetrad scar on proximal polar area, subsequatorial circumpolar canal, thickened exinous band on equatorial region. Monoporate with a circular distal pore (12–15 µm in diameter). Spherical, ovoid, or corn-shaped; circular in equatorial section. **Exine**: Tectate, thicker on the equatorial area, thinner on cryptopore, trifid tetrad scar, and subequatorial canal. Two-layered; intexine thin and continuous, occasionally showing a small trilete mark. Exoexine 1-2 µm thick, with distinct zones and annular bands of thickening. **Nexine** absent; sexine two-layered with tectate and baculite layers. **Infratectal Sculpture**: Baculite or verrucose, creating various patterns visible under a microscope. **Ornamentation**: Small pits forming continuous bands around the equator, granulose or microreticulate patterns over the rest of the exoexine. [13;6].	*Classopollis classoides* Pflug 1953, emend. Pocock and Jansonius 1961 [13] (Fig. 9: D, D’. L, M.); (Fig. 11; C-E). **Structure**: Anisopolar, amb circular, composed in polar direction. **Key Features**: Circular cryptopore on the distal polar area, no tetrad mark observed, central cavity that might have a biological role. **Exine**: 1–2 µm thick in the equatorial region, thinning towards the pole. **Proximal Pole**: Usually has a triangular area.
*Classopollis simplex* (Danzé-Corsin and Laveine 1963) Reiser and Williams 1969 [35]	**Grains**: Central body with reticulate (net-like) patterns and a surrounding annulus (ring) [56].	*Classopollis simplex* (Danzé-Corsin and Laveine 1963) Reiser and Williams 1969 [35] (Fig 7: A-G; Fig 9: B). **Grains**: **Structure**: Anisopolar, amb circular. **Exine**: 1–2 µm thick in the equatorial region, thinning towards the pole. **Proximal Pole**: Usually has a circular area. **Distal Pole**: Rarely characterized by the presence of a circular pore. **Central Cavity**: Present without a tetrad mark.
*Classopollis brasiliensis* Herngreen 1975 [33]	Pollen grains include a central body with a reticulate (net-like) pattern and a surrounding annulus [ring). [33].	*Classopollis brasiliensis* Herngreen 1975 [33] (Fig. 8: A-D). **Structure**: Anisopolar, amb rounded to oval. **Ornamentation**: Striated, connecting the poles. **Proximal Pole**: Usually has an oval area. **Central Cavity**: Present without a tetrad mark, surrounded by striations.
*Classopollis torrosus* Reissinger 1950; Couper 1958	**Pollen Structure**: Round or oval amb, flattened acorn-shaped in lateral view. **Key Features**: Vestigial tetrad markings (triangular or triradiate scars) on the proximal face, distal operculum (~20 µm in diameter). **Exine**: About 2 µm thick in polar areas, thickened in a narrow equatorial zone (appears as an equatorial rim in polar view). Rim ornamented with shallow, radially elongated pits, remainder of the exine scabrate. **Dimensions**: Mean 28 µm, polar diameter about 25 µm. [12].	*Classopollis* sp. (Fig 7: I-K and 8: G, J, N). *Classopollis torrosus* Reissinger 1950 [39]; Couper 1958 **Structure**: Anisopolar, amb circular. **Key Features**: Pore aperture in proximal pole, ring-shaped transition zone between distal hemisphere and equatorial band. Tetrahedral and individual pollen. **Types**: Some grains are monoporate, and amb circular to subcircular. **Exine**: 2–4 µm thick. **Sculpture**: Minute granules and small folds in some specimens.
*Classopollis meyeriana* (Klaus 1960) de Jersey 1973	**Spore Structure**: Smooth to indistinct and finely infrapunctate. **Key Features**: Proximal dehiscence triangle with straight to slightly concave sides (length 2/3 to 1/1 of spore radius), narrow ring tenuitas close to the equatorial outline (4–5 µm distance in medium-sized grains), distal tenuitas with irregular circular contour (diameter about 1/2 spore radius), slight polygonal deformation. **Pollen Structure**: Monad or regular tetrad. **Key Features**: Polar pore and subequatorial annulicolpus (14:56).	*Classopollis meyeriana* (Klaus 1960) [14] de Jersey 1973 (Fig 10; L) **Exine**: Two-layered, with the inner exine thicker than the outer one. **Sculpture**: Levigate. **Key Features**: Cryptopore, tetrad mark, and canal observed. Central cavity present without a tetrad mark.

The botanical affinity of *Classopollis* Pflug 1953, has been studied based on a matrix containing conifer male cones of *Brachyphyllum scotti* Kendall 1948 [57] from the matrix containing the conifer male cone of *Brachyphyllum scotti* Kendall from the Scottish Lias [58]. There are similar pollen grains that were also found in male cones linked to *Pagiophyllum connivens* Kendal from the Bajocian of Yorkshire, England [58–59], despite the *in situ* absence of these pollen grains [6] (Figs 12 and 13).

*Classopollis* Pflug 1953 was characterized by sporangia of *Cheirolepis muensteri* (Schenk) Schimper (=*Hirmerella muensteri* Hörhammer 1933 [60] Jung collected from Rhaeto-Liassic strata of south Wales, with considerations on uncertainties regarding the affiliation of *Pagiophyllum connivens* Kendall to Araucariaceae, which exhibited pollen resembling *Cheirolepis* [6].

The botanical relationship between the family Cheirolepidiaceae and the genera *Masculostrobus* Seward, and potentially with Brachyphyllum *Cheirolepis*, *Masculostrobus*, and *Pagiophyllum*, was highlighted [61]. *Classopollis* Pflug 1953, pollen was also found in male cones of *Tomaxellia biforme* Archangelsky 1963 [49], from the Lower Cretaceous of Argentina, suggesting a botanical connection with the genus *Tomaxellia* of the Family Cheirolepidiaceae. However, pollen grains of the species *Inaperturopollenites limbatus* Balme 1957 [12], were discovered in cones of *Brachyphyllum irregulare* Archangelsky and Gamerro 1967 [62]. The *Classopollis* Pflug 1953 found in the Obayied Field, shows significant similarity to the documented lower Cretaceous sporomorphs of the Bougaz-1 well, northeast Sinai [63]. The assumption of the extent of the same parent plant producing the *Classopollis* Pflug 1953, in the currently studied areas could be important to consider.

### 5.1. Interpretations of the proposed paleo-heat events during the Mesozoic era

#### 5.1.1. The similarity of the *Classopollis* Pflug 1953 in two studied fields for the palaeoecology inferences.

The section discusses the probability of proposed paleo-heated events related to the findings of the *Classopollis* Pflug 1953, in different coastal and non-coastal areas. The newly observed SEM of the current species of *Classopollis* Pflug 1953 [11] is not being globally distributed, which could be of endemic features. The plants that produce *Classopollis* Pflug 1953, provide hints about the environment where Cheirolepidiaceae first thrived in Egypt. The record of *Classopollis* Pflug 1953, in tetrads in the Obayied samples implies proximity to the source area [64], and the record of *Classopollis* spp. on the surface of cuticle in the October Field also suggests a near source of vegetation.

*Classopollis* Pflug 1953, *Ephedripites* Bolchovitina and Potonié 1958 have characterized arid vegetation during warm summers [56; 8]. The high representation of *Classopollis* Pflug 1953 and the rare or absence of *Dicheiropollis etruscus* Trevisan 1971 in the study wells could indicate similar climates across different regions [8]. The dominance of the *Classopollis* Pflug 1953, and the absence of the *Dicheiropollis etruscus* Trevisan 1971, could infer that paleoecological conditions prevent the production of the *Dicheiropollis etruscus* Trevisan 1971. This assumption needs further studies.

The presence of gymnosperm pollen, including *Classopollis* Pflug 1953, and nonaperturate pollen, such as *Araucariacites* Cookson and Couper 1953 and *Balmeiopsis* Archangelsky 1979*,* in association with *Ephedripites* Bolchovitina and Potonié 1958, reflects that the Malha Formation may have been sourced by coastal vegetation [56–66]. This dominance of gymnosperm pollen composed of circular and inaperturate types from the October Field in the Gulf of Suez of the studied samples closely resembles the palynological content of the Malha Formation, which was considered equivalent to the uppermost unit of the Nubia sandstone. The presence of the *Classopollis* Pflug 1953, in the studied samples from the Nubia sandstone can indicate the oldest occurrences of *Classopollis* Pflug 1953, to be of the late Triassic age based on the other associated microflora. The occurrences of the *Classopollis* Pflug 1953 [11] are associated with Triassic microflora such as *Aratrisporites* sp., *Leiotriletes* sp., *Ovalipollis ovalis* (Krutzsch] Scheuring 1970, *Praecolpatites sinuosus* Balme and Hennelly 1956, *Tricolpites* sp., and *Kraeuselisporites cuspidus* Balme 1963 (Fig 14; A-F).

In the studied samples of the Obayied Field, *Classopollis* Pflug 1953, *Ephedraoid* Bolchovitina and Potonié 1958 were observed in the OBA-D26 Well. This agrees with the facies recorded by [67], who studied the Arab “D” unit in Qatar in the eastern Arabian Peninsula and assigned it to the Kimmeridgian age. The Arab D member was proposed to have been deposited in shallow water of middle shelf depth (30–50 m) under arid to semiarid climatic conditions, as deduced from the presence of *Classopollis* Pflug 1953 and the capping anhydrite. There was observation of the replacement of *Classopollis* Pflug 1953, by *Araucariacites* Cookson & Couper 1953, and ferns reflect a change from dry to wet conditions with noticed growth of anhydrite nodules, and in beds overlying the evaporites, there was a conspicuous increase in flora associated with humid conditions [68]. The *Classopollis* Pflug 1953, recorded from the October Field, are observed to be from deposits rich in evaporites and gypsum. The *Classopollis* Pflug 1953 was produced by shrubs that tolerated semiarid conditions, and it could have occurred in the desert basins, but the shrubs may have also lived in xeric uplands [6].

The recorded *Classopollis* Pflug 1953, from the two fields, is presumed to represent the arid climate belt around the Arabian Plate. The paleontological and paleoecological significance of the early Jurassic beds containing *Classopollis chateaunovi* Reyre 1970 [5] assemblage-subzone produced high percentages of *Classopollis* Pflug 1953 can indicate the arid conditions [69]. This *Classopollis chateaunovi* Reyre 1970 [5] is well represented in the current studied samples. The thermophilic conifers Cheirolepidiaceae and their distinctive pollen *Classopollis* Pflug 1953, are the most abundant representatives of the xerophytic flora [70]. This suggests that the Cheirolepidiaceae played a role in the coastal and non-coastal ecosystems of the Tethys, providing new insights into their ecological characteristics and adaptive strategies.

#### 5.1.2. The arid paleoclimate linked to paleo-swamp vegetation.

The pollen of Cheirolepidiaceae plants has been suggested to identify the characteristic xerophytic, drought-resistant, and thermophilic plants found in the shallow marine deposits of the Tendaguru Beds in southeast Tanzania, dating back to the Upper Jurassic and Lower Cretaceous [69]. The palynological study by [71] from the Obayied Field shows similar palynological data to the currently studied samples. This indicates that the same paleovegetation cover contained plants producing *Classopollis* Pflug 1953*,* such as *Classopollis classoides* Pflug 1953 [11].

The plants producing *Classopollis* Pflug 1953 pollen reconstructed from the present study could represent coastal vegetation belts. This belt of plants was characterized by an arid climate phase, possibly connected to the warm and arid climate phase that occurred in the latest Jurassic of Western Europe [72]. It is suggested that the *Classopollis* Pflug 1953, could be deposited in the Late Jurassic into lower Cretaceous sedimentation processes [71] in the Alam El Bueib Member [73–74]. *Classopollis* Pflug 1953, and other circumpolloid forms are characteristic of Late Triassic and Jurassic rocks worldwide and are present until the end of the Cretaceous period and even into the Paleogene [75].

The high abundance of pollen from the genus *Classopollis* Pflug 1953, is indicative of Lower Cretaceous regions in Brazil, West Africa, Argentina, and South Africa, suggesting that plants producing this pollen grew on seashores [67].

The presence of abundant *Classopollis* Pflug 1953 is linked to evaporites, salts, and red bed deposits [72], as well as xeromorphic wood and leaf macrofossils with Cheirolepidiacean [8]. The vegetation in northeastern Brazil during the Aptian period was well-suited to semi-arid and arid climates, indicated by the prevalence of warmth-loving conifers from the Cheirolepidiaceae family and their pollen, *Classopollis* Pflug 1953, in upper Aptian rocks across most of the continental margin basins of the South Atlantic [76, 77]. The presence of abundant thermophilic xerophytic pollen like *Classopollis* Pflug 1953, and hygrophilous fern spores suggests that the nearby continent had a semi-arid to arid environment with limited swamp vegetation [78]. The record of the *Classopollis* Pflug 1953, in the October and Obayied study fields, can indicate that they might be of lagoon and shoreface depositional paleoenvironments. The highest comparative abundances of *Classopollis* Pflug 1953, were recorded from delta front, lagoon, and shoreface depositional environments marked by high mud fractions [79]. The salty environment near the coastal areas containing Cheirolepidiaceae produced *Classopollis* Pflug 1953, indicates high temperatures [80] in both October and Obayied fields of the different regions of northern Egypt.

The similarity between the October and Obayied fields containing the *Classopollis* Pflug 1953, assemblage suggests diverse ecological conditions within the Cheirolepidiaceae. This similarity between the *Classopollis* Pflug 1953 recorded from both the Obayied Field and October Field, infers the same phytogeographic cover. This family encompasses a wide range of habitats, from small shrubs to trees, which control various environmental niches. The resemblance between the two fields highlights the distribution of Cheirolepidiaceae in the coastal regions covering both areas. The discovery of thermophilous flora belonging to Cheirolepidiaceae implies their role in establishing a specific zone in the coastal areas of the Tethys. *Classopollis classoides* Fensome 1983 [34] was considered thermophilic and is a reliable proxy for hot/warm climatic conditions [81]. The occurrence of the thermophilous genus *Classopollis* Pflug 1953, indicates warm climatic conditions [82].

The reconstructed paleophytogeographic cover studied from the different areas is presumed to be dominated by the Cheirolepidiaceae plant, which produces *Classopollis* Pflug 1953. The similarity between the *Classopollis* Pflug 1953 from the two fields indicates the likely presence of an extended landmass between the Gulf areas and the Northwestern Desert areas. This could indicate a cosmopolitan habitat for the plants producing the pollen of the genus *Classopollis* Pflug 1953*,* which shows the uniformity of the paleoclimate for the regions that were reported to contain this pollen, especially for the coastal areas of Tethys under dry climatic conditions [6].

The West African *Classopollis* Pflug 1953 lineages show a gradual increase in size from 22 to 28 µm in the Aptian-Albian (*Classopollis classoides sensu* Jardiné and Magloire 1965 [83] to around 50 µm (*Classopollis* sp., of Jardiné and Magloire 1965 =  *C*. *brasiliensis*) in the Cenomanian [84]. In the specimens studied, there is a noticeable increase in the size of *Classopollis* Pflug 1953 pollen, and the inner opening area is observed in detail from several species for the first time (Fig 10; H-L).

The *Classopollis* Pflug 1953 pollen recorded from the October Field could represent the earliest appearance of *Classopollis* Pflug 1953 in Egypt, and it is proposed to be part of Gondwanan Cheirolepid diversification [3].

The zone of Cheirolepidiaceae pollen taxa recorded from the October Field should belong to the equivalent part of the Malha Formation, as concluded by [66]. Moreover, this zone of Cheirolepidiaceae pollen, which extended to the Obayied Field, is of the same age (Fig 6). These similar findings reflect that the Nubia sandstone’s studied part bearing the *Classopollis* Pflug 1953, [11] spike can be regarded as a marker for the stratigraphic uses for containing the Cheirolepidiaceae family. The correlation between the studied interval of the October Field and the same interval of the Obayied Field is suggested to be of the Mesozoic ages, despite *Classopollis* Pflug 1953 ( = *Brachyphyllum*) having been assigned from the Upper Permian to the Upper Cretaceous [85–86].

### 5.2. Stratigraphic range of sediments containing the *Classopollis* Pflug 1953 in the present study

The genus *Classopollis* Pflug 1953 has worldwide occurrences in Upper Triassic-Turonian strata [6], despite some species (*Classopollis belloyensis*) being described from the Upper Permian of Canada [13], which rejected the latter [62]. In Egypt, the record of *Classopollis classoides* Fensome 1983 [34] *is* assigned to Bajocian-Bathonian [87], the Late Barremian-Middle Aptian [88–89], and Aptian-Turonian [90], Middle-Late Albian [91], and lower Cenomanian [92]. *Classopollis brasiliensis* Herngreen 1975 [33] was recorded from Aptian–Cenomanian [93] and Cenomanian [94]. *Classopollis torrosus* Burger 1965 [15] was recorded from Late Bajocian-Bathonian [95], Bathonian [96], lower Kimmeridgian-Middle Kimmeridgian [90], Bathonian-Hauterivian [97], Bajocian-Bathonian [98], Neocomian [99], Neocomian–lower Aptian [100], and Albian-lower Cenomanian [101].

In the present study, the *Classopollis* Pflug 1953 assemblage recorded from the Obayied Field is obtained from Barremian-Aptian microflora, while that recorded from the October Field is presumed to have been obtained from the latest Triassic ages. These two *Classopollis* Pflug 1953 assemblages recorded from the two fields [102] are presumed to represent the older record from the October Field (latest Triassic age) and the younger age from the Obayied Field (Barremian-Aptian age). Therefore, the genus *Classopollis* Pflug 1953 is proposed to have started to appear in Egypt during the latest Triassic and continued until the Aptian age based on the currently recorded fossil pollen of this *Classopollis* Pflug 1953.

The group of plants producing *Classopollis* Pflug 1953 is believed to have become extinct in the Upper Cretaceous despite their male cones resembling those of the Araucariaceae [6].

The recorded *Classopollis* Pflug 1953, from the four studied wells explains the evolutionary trend of this genus from the latest Triassic to Barremian-Aptian age. This indicates the continued flourishing of this plant during the early Mesozoic ages, which infers that the plant-producing *Classopollis* Pflug 1953, adapted to the arid climate zone of Northern Gondwana.

## Conclusions

The palynological study suggests the flourishing of the Cheirolepidiaceae in the Tethys Mesozoic coastal and non-coastal areas in Egypt. This indicates that paleovegetation zones composed of Cheirolepidiaceae may have had broader ecological implications during the Mesozoic era. The study suggests that the thermophilic flora of Cheirolepidiaceae played a role in establishing coastal and desert vegetational zones in the paleo-areas of the Tethys, which reflect the survival of the Cheirolepidiaceae family in the desert and coastal marshes of the Gulf regions during the Mesozoic. The *Classopollis* Pflug 1953 assemblage peak is suggestive of stratigraphic marker ecological and climatic conditions in the Mesozoic of Egypt. The *Classopollis* have suffered during the latest Triassic to the Barremian–Aptian ages in Egypt to reflect the strong ecological adapting of their parent plants under the arid climate conditions.
